# Reduction of Interface State Density in 4H-SiC MOS Capacitors Modified by ALD-Deposited Interlayers

**DOI:** 10.3390/nano15070555

**Published:** 2025-04-05

**Authors:** Zhenyu Wang, Zhaopeng Bai, Yunduo Guo, Chengxi Ding, Qimin Huang, Lin Gu, Yi Shen, Qingchun Zhang, Hongping Ma

**Affiliations:** 1 Institute of Wide Bandgap Semiconductors and Future Lighting, Academy for Engineering & Technology, Fudan University, Shanghai 200433, China; 22210860082@m.fudan.edu.cn (Z.W.); 22210860001@m.fudan.edu.cn (Y.G.); cqding23@m.fudan.edu.cn (C.D.); 22210860073@m.fudan.edu.cn (Q.H.); 24110860041@m.fudan.edu.cn (L.G.); shenyi21@m.fudan.edu.cn (Y.S.); qingchun_zhang@fudan.edu.cn (Q.Z.); 2 Shanghai Research Center for Silicon Carbide Power Devices Engineering & Technology, Fudan University, Shanghai 200433, China; 3 Institute of Wide Bandgap Semiconductor Materials and Devices, Research Institute of Fudan University in Ningbo, Ningbo 315327, China

**Keywords:** 4H-SiC, SiC/SiO_2_ interfaces, electrical properties, capacitors

## Abstract

This study proposed an innovative method for growing gate oxide on silicon carbide (SiC), where silicon oxide (SiO_2_) was fabricated on a deposited Al_2_O_3_ layer, achieving high quality gate oxide. A thin Al_2_O_3_ passivation layer was deposited via atomic layer deposition (ALD), followed by Si deposition and reoxidation to fabricate a MOS structure. The effects of different ALD growth cycles on the interface chemical composition, trap density, breakdown characteristics, and bias stress stability of the MOS capacitors were systematically investigated. X-ray photoelectron spectroscopy (XPS) analyses revealed that an ALD Al_2_O_3_ passivation layer with 10 growth cycles effectively suppresses the formation of the proportion of Si-O_x_C_y_ bonds. Additionally, the SiO_2_/Al_2_O_3_/SiC gate stack with 10 ALD growth cycles exhibited optimal electrical properties, including a minimum interface state density (*D*_it_) value of 3 × 10^11^ cm^−2^ eV^−1^ and a breakdown field (*E*_bd_) of 10.9 MV/cm. We also systematically analyzed the bias stress stability of the capacitors at room temperature and elevated temperatures. Analysis of flat-band voltage (Δ*V*_fb_) and midgap voltage (Δ*V*_mg_) hysteresis after high-temperature positive and negative bias stress demonstrated that incorporating a thin Al_2_O_3_ layer at the interface is the key factor in enhancing the stability of *V*_fb_ and midgap voltage *V*_mg_.

## 1. Introduction

Due to its high breakdown electric field and thermal conductivity, SiC has emerged as one of the most promising wide-bandgap semiconductors in power electronics [1,2,3]. However, despite the high bulk electron mobility of 4H-SiC MOSFETs, their performance remains constrained by defects at the SiO_2_/SiC interface, particularly carbon-related defects resulting from the oxidation of SiC [4,5,6]. Consequently, the gate oxide performance of SiC MOSFETs remains suboptimal, with field effect mobility significantly lower than the theoretical potential of SiC material, notable threshold voltage instability, and high interface trap densities. These limitations stem primarily from poor MOS interface quality, where interfacial carbon clusters, silicon and carbon vacancies, and oxide charges significantly impede carrier transport [7,8,9]. While commercial SiC MOSFETs operating at 1.2–1.7 kV have been realized, further improvements in interface quality are essential to fully exploit SiC’s high voltage capability for future 3.3–6.5 kV devices.

To enhance the channel mobility of SiC MOSFETs, various passivation techniques have been employed over the past few decades. Post-oxidation annealing (POA) processes using nitrogen, phosphorus, and other elements have elevated channel mobility [10,11,12,13,14,15,16,17]. One strategy involves initially depositing a layer of Si, followed by low-temperature oxidation to construct a theoretically carbon-cluster-free SiC/SiO_2_ interface, and subsequently annealing to obtain a high-quality SiO_2_ thin film [18]. However, research on this method remains limited. Some studies have indicated that the final oxidation process may lead to the formation of a SiO_2_/Si/SiC structure, exhibiting poor *C*–*V* characteristics, thereby necessitating further investigation [19].

Introducing high dielectric constant (high-k) materials is a potential solution [20,21,22]. Nevertheless, electrical tests of high-k/SiC MOS devices exhibit significant frequency dispersion [23,24,25]. Several studies have attempted to use Al_2_O_3_/SiO_2_/SiC gate stacks to reduce interface defects, achieving some success, but their *C*–*V* curves remain suboptimal, with considerable hysteresis and frequency dispersion [26,27].

Theoretical calculations have shown that carbon defects readily form and stabilize at the SiO_2_/SiC interface [11], while experimental studies have found that inhibiting the oxidation of the SiC surface can effectively reduce the *D*_it_ [12]. Therefore, suppressing the oxidation of the SiC substrate during device fabrication is crucial for minimizing interfacial carbon defects and enhancing interface quality [28]. The dense Al_2_O_3_ film effectively inhibits the diffusion of oxygen atoms, particularly gaseous oxygen, thereby preventing oxidation of the underlying SiC [29]. In addition, Jinyu et al. proposed that Al_2_O_3_ can serve as a passivation layer to improve the interface performance of ErSmO/InP MOS devices, further supporting the strategy of reducing interface defects [30]. Currently, most research involves depositing Al_2_O_3_ onto ultra-thin thermally oxidized SiO_2_ [27,31], but no studies have explored the formation SiO_2_ through thermal oxidation on ultra-thin deposited Al_2_O_3_.

In light of these findings, we propose a novel gate oxide fabrication process: first, a thin layer of Al_2_O_3_ was deposited using ALD to protect the SiC surface with its exceptional density. Subsequently, Si was deposited and re-oxidized. Leveraging this thin Al_2_O_3_ layer, we achieved a high-quality gate oxide layer without the need for annealing. Using this method, we successfully improved the interface properties of SiC MOS capacitors and enhanced the stability of the gate flat-band voltage. The electrical performance of the SiC MOS capacitors was assessed through measurements such as *I*–*V* characteristics, HF-QS *C*–*V*, and *C*–*V* tests under positive and negative bias stress. Additionally, AFM was employed to study the morphology of the SiO_2_ surface, and XPS was utilized to analyze the chemical bonding at the SiC/SiO_2_ interface, revealing the effectiveness of our process from a microscopic perspective.

## 2. Experimental Methods

### 2.1. Fabrication of SiC MOS Capacitors

**Figure 1:**
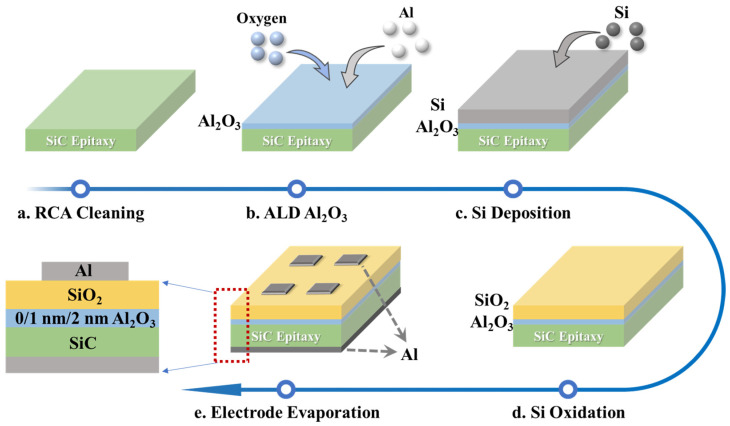
Fabrication process flow of the SiC MOS capacitors.

The substrates were n-type 4H-SiC (0001). The epitaxial layer had a doping concentration equal to 1 × 10^16^ cm^−3^ and a thickness of 5 μm. As shown in Figure 1, the experimental procedure was as follows:

(a) The SiC epitaxial wafers were subjected to RCA cleaning to remove surface impurities, ensuring that the wafer surface was clean for the smooth progression of subsequent processes.
(b) Al_2_O_3_ was deposited on the epitaxial wafer surface using ALD technology with 10 and 20 cycles, while a sample without Al_2_O_3_ deposition was prepared as a control group.
(c) A 20 nm thick Si layer was grown on the prepared three groups of samples in a conventional plasma-enhanced chemical vapor deposition (PECVD) system. The Si film was deposited using SiH_4_ gas highly diluted with H_4._ The PECVD system operated at a radio frequency of 13.56 MHz, a substrate temperature of 250 °C, and an r.f. power of 50 W.
(d) The samples underwent wet oxidation at 1100 °C for 2 h, during which the PECVD-grown silicon films were fully oxidized into SiO_2_ layers, with consistent silicon growth and thermal oxidation conditions maintained across all samples to ensure experimental uniformity. The samples were classified as follows: SiO_2_/SiC-A sample without Al_2_O_3_ deposition; SiO_2_/1 nm-Al_2_O_3_/SiC-A sample with 10 cycles of Al_2_O_3_ passivation layers; SiO_2_/2 nm-Al_2_O_3_/SiC-A sample with 20 cycles of Al_2_O_3_ passivation layers.
(e) After coating a layer of photoresist on the front side of the sample, hydrofluoric acid was used to remove the excess SiO_2_ from the back side of the samples. The photoresist was then removed, and square electrodes, 200 μm in width and 200 nm in thickness, were deposited on the front side using electron beam evaporation. Additionally, a 200 μm thick aluminum electrode was also deposited on the back side of the sample.

### 2.2. Characterization

The samples’ surface roughness (*R*q) and morphology were examined using a Bruker BRK0003 atomic force microscope (AFM) over areas of the sample measuring 5 × 5 μm^2^. Multiple measurements (*n* = 10) were performed on different areas of each sample, and the most reliable roughness values were determined through statistical analysis for data presentation. The surface chemical states were analyzed using an Escalab 250Xi XPS system from Thermo Scientific equipped with a monochromatic Al-Kα X-ray source at 1486.6 eV and a power of 150 W. Before the XPS analysis, the SiO_2_ film on the SiC epitaxy was etched to a thickness of approximately 5 nm using a 1% HF buffered solution.

The electrical properties of the SiC/SiO_2_ capacitors were characterized using a TS2000-HP probe station and a Keithley 4200A-S semiconductor analyzer. The insulation and breakdown characteristics of the oxide layer were evaluated by recording the current–voltage (*I*–*V*) curves at room temperature. The *D*_it_ was extracted using the high–low frequency method within an energy range 0.2–0.6 eV below the conduction band edge. The *C*–*V* curves were measured at 300 K using high-frequency (1 MHz) and quasi-static methods. The voltage stabilities were evaluated utilizing *V*_fb_ hysteresis after alternate positive bias stress (PBS) and negative bias stress (NBS) at different temperatures (300, 350, 400, and 450 K). The *C*–*V* curves were swept from +20 to −20 V after the PBS (+2.5 MV cm^−1^) for 600 s. Following the same procedure, the *C*–*V* curves were swept from −20 to +20 V after the NBS (−2.5 MV cm^−1^) for 600 s. The *V*_fb_/midgap voltage (*V*_mg_) hysteresis was obtained from the shift between *V*_fb_ and *V*_mg_ after PBS and NBS.

## 3. Results and Discussion

### 3.1. Interfacial Qualities and Characteristics of the SiO_2_ Films

**Figure 2:**
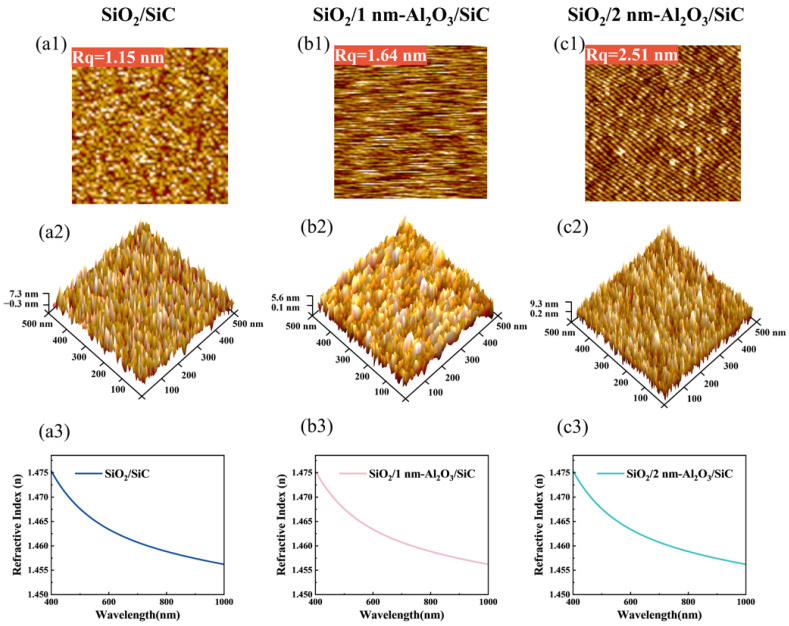
Both 2D and 3D AFM images within the area of 5 × 5 μm^2^ for (**a1**,**a2**) SiO_2_/SiC, (**b1**,**b2**) SiO_2_/1 nm-Al_2_O_3_/SiC, and (**c1**,**c2**) SiO_2_/2 nm-Al_2_O_3_/SiC. SE results for (**a3**) SiO_2_/SiC, (**b3**) SiO_2_/1 nm-Al_2_O_3_/SiC, and (**c3**) SiO_2_/2 nm-Al_2_O_3_/SiC.

The surface roughness of the MOS capacitor was characterized using AFM with a scanning area of 5 × 5 μm^2^. The AFM topographic image was shown in Figure 2, and the root mean square roughness values of the gate dielectric surface were calculated to be 1.15 nm, 1.64 nm, and 2.51 nm, respectively. When the passivation cycle reaches 10 times, the surface roughness of the SiO_2_ layer slightly increases to 1.64 nm compared to the control sample. It is noteworthy that the surface roughness increases significantly to 2.51 nm as the passivation interlayer thickens. This increase in roughness can be attributed to the additional ALD process step, which introduces more interfaces and potentially creates more surface irregularities during the fabrication process. However, this increase in surface roughness does not negatively impact the passivation effectiveness of the Al_2_O_3_ interlayer, as evidenced by our subsequent electrical measurements, which demonstrate that the samples with an Al_2_O_3_ passivation layer exhibit lower interface defect density and wider barrier height.

XPS was used to analyze the chemical bonds at the SiO_2_/SiC interface. The C 1s peak, attributed to adventitious carbon, is typically fixed at approximately 284.8 eV upon calibration of the measurements using a charge neutralizer.

**Figure 3:**
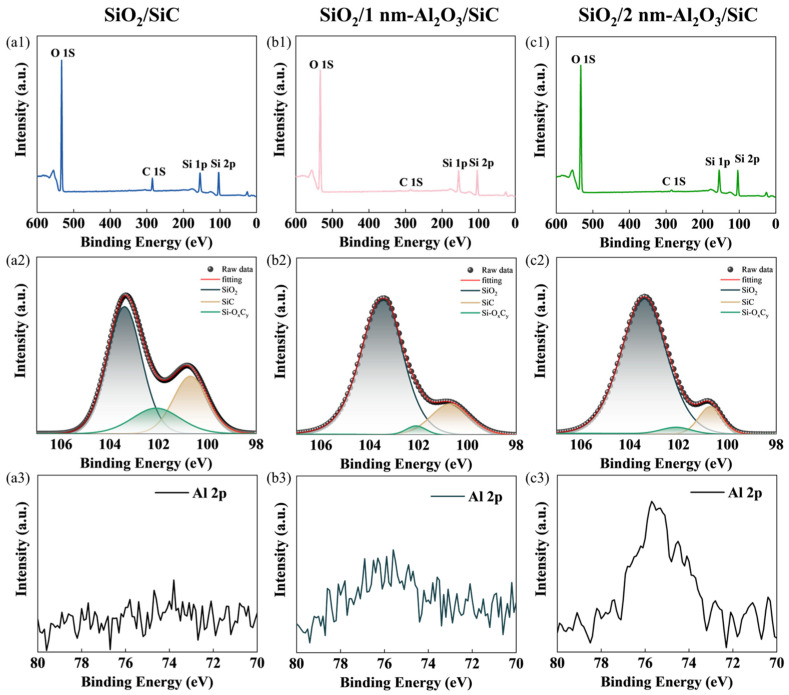
Acquired XPS survey spectrum for (**a1**) SiO_2_/SiC; (**b1**) SiO_2_/1 nm-Al_2_O_3_/SiC and (**c1**) SiO_2_/2 nm-Al_2_O_3_/SiC. Deconvolution of the Si 2p core levels for (**a2**) SiO_2_/SiC; (**b2**) SiO_2_/1 nm-Al_2_O_3_/SiC and (**c2**) SiO_2_/2 nm-Al_2_O_3_/SiC. Obtained XPS spectra of the Al 2p core levels for (**a3**) SiO_2_/SiC; (**b3**) SiO_2_/1 nm-Al_2_O_3_/SiC and (**c3**) SiO_2_/2 nm-Al_2_O_3_/SiC.

Figure 3 compares the XPS results of the SiC/SiO_2_ interface with different Al_2_O_3_ passivation layers. Two weak Al 2p peaks were detected in both Figure 3(b3,c3) compared to Figure 3(a3). The peak in SiO_2_/1 nm-Al_2_O_3_/SiC with 10 ALD growth cycles in Figure 3(b3) is slightly weaker, while the peak in SiO_2_/2 nm-Al_2_O_3_/SiC with 20 ALD growth cycles in Figure 3(c3) is slightly stronger.

In the Si 2p spectrum, the binding energies are 100.7, 101.7, and 103.1 eV, corresponding to Si–C, Si–O_x_C_y_, and Si–O bonds, respectively [32,33,34]. The Si 2p spectrum shows a decrease in the SiOxCy components after the passivation layer was added, as shown in Figure 3(b2,c2). These observations indicate that it is unrealistic to prevent oxidation of the SiC substrate solely by temperature control, as low-temperature oxidation may still introduce some issues to the interface properties. However, an appropriate Al_2_O_3_ passivation cycle can effectively suppress defect formation.

**Figure 4:**
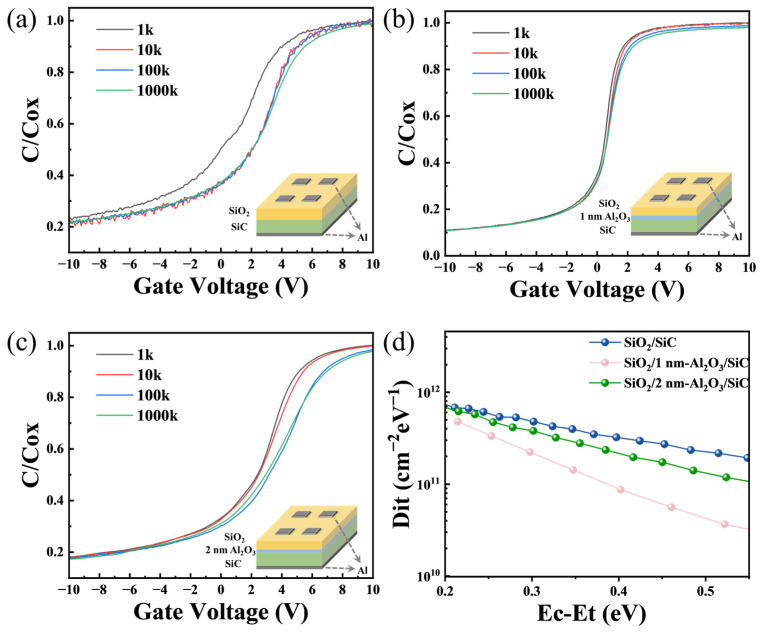
(**a**) Obtained *C*–*V* curves for the SiO_2_/SiC at different test frequencies; (**b**) Obtained *C*–*V* curves for the SiO_2_/1 nm-Al_2_O_3_/SiC at different test frequencies; (**c**) Obtained *C*–*V* curves for the SiO_2_/2 nm-Al_2_O_3_/SiC at different test frequencies; (**d**) Distribution of *D*_it_ vs. energy level for SiO_2_/SiC, SiO_2_/1 nm-Al_2_O_3_/SiC, and SiO_2_/2 nm-Al_2_O_3_/SiC.

As shown in Figure 4, to study the effect of different passivation growth cycles on the capacitance parameters of SiC/SiO_2_ capacitors, the *C*–*V* characteristics were measured in the frequency range from 1 kHz to 1 MHz. The experiment revealed that each sample exhibited varying degrees of frequency dispersion. Adding 10 cycles of Al_2_O_3_ passivation layer helps to suppress the generation of interface defects, allowing most carriers to accumulate effectively on the semiconductor surface, thereby improving the dielectric performance of the SiC MOS capacitors. When the passivation cycle reaches 20c, SiO_2_/2 nm-Al_2_O_3_/SiC exhibits significant frequency dispersion. To quantitatively analyze the impact of different passivation layer thicknesses on the SiO_2_/SiC interface characteristics, we extracted the values of *D*_it_ for each sample, as shown in Figure 4d. The results indicate that around *E_c_* − *E_t_* ≈ 0.3 eV, the *D*_it_ values for the three samples are approximately: 5 × 10^11^, 2 × 10^11^, and 4 × 10^11^ cm^−2^ eV^−1^, respectively. Compared to the structure without a passivation layer, the *D*_it_ at various energy levels is reduced after adding the Al_2_O_3_ passivation layer, especially around the trap energy level of approximately 0.6 eV, where a more significant reduction in *D*_it_ is observed. This demonstrates that the addition of the passivation layer effectively reduces the interface state density, thereby improving the quality of the interface, with the SiO_2_/1 nm-Al_2_O_3_/SiC structure exhibiting lower *D*_it_ values than the SiO_2_/SiC structure; moreover, compared to the previously reported Al_2_O_3_/SiC structures [35], our composite structure also achieves a significant reduction in *D*_it_ values, further confirming the effectiveness of this passivation approach.

However, in the MOS structure with the Al_2_O_3_ passivation layer, the *D*_it_ value for SiO_2_/2 nm-Al_2_O_3_/SiC configuration is obser ved to be higher than that for the SiO_2_/1 nm-Al_2_O_3_/SiC configuration. This deterioration in interface quality with increasing Al_2_O_3_ thickness (from 1 nm to 2 nm) can be attributed to the underlying mechanisms governing interface formation. As reported by Boan et al. [26], when gate materials are directly deposited on SiC using ALD techniques, the resulting interface typically exhibits significantly higher *D*_it_ values compared to thermally grown oxides. Their research revealed that Al_2_O_3_/SiC interfaces formed through direct deposition generally exhibit poor interface quality. To address this issue, researchers have developed two main approaches: (1) Applying post-deposition annealing treatments to improve the electrical properties of the ALD-deposited dielectric layer and its interface with SiC [36,37]; (2) Adopting a multilayer structure where SiC is first thermally oxidized to form a SiO_2_ layer before depositing high-K materials (such as HfO_2_/Al_2_O_3_) on top, creating a high-K material/thermally oxidized SiO_2_/SiC stack that significantly reduces interface trap densities [27,38]. Based on these findings, we propose that as the Al_2_O_3_ layer thickness increases from 1 nm to 2 nm, its functional role shifts from primarily acting as a passivation layer to resembling a directly ALD-deposited gate oxide in direct contact with SiC. At 1 nm thickness, the Al_2_O_3_ layer effectively passivates interface traps while preserving the advantageous properties of the underlying thermally grown SiO_2_/SiC interface. However, at 2 nm thickness, the Al_2_O_3_ layer begins to dominate the interface characteristics, leading to properties that closely resemble those of direct ALD-deposited Al_2_O_3_/SiC structures, which inherently exhibit higher *D*_it_ values. This thickness-dependent transition in interface behavior accounts for the observed increase in *D*_it_ values despite the increased physical thickness of the passivation layer. Consequently, the careful optimization of the Al_2_O_3_ passivation layer thickness is crucial to prevent the Al_2_O_3_ layer from transitioning from a passivation effect to a layered structure.

**Figure 5:**
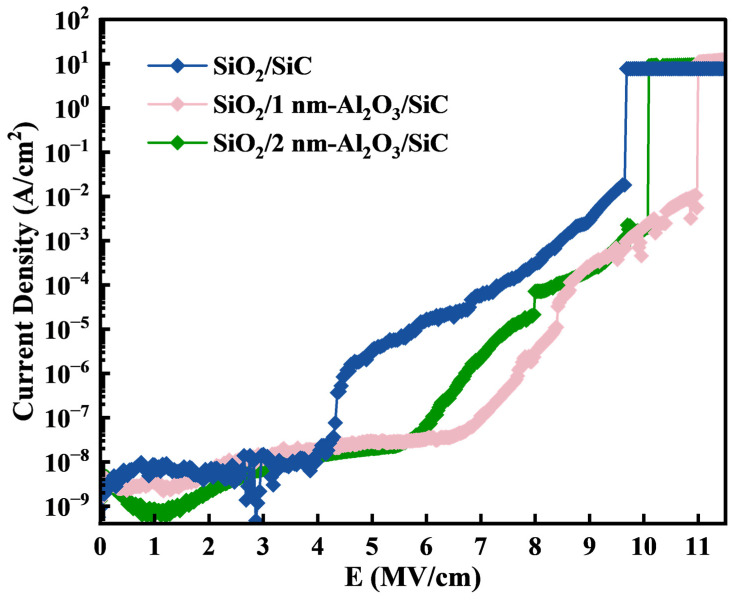
Room temperature *J*–*E* curves for SiC MOS capacitors based on samples with different Al_2_O_3_ growth cycles.

To study the effect of the Al_2_O_3_ passivation layer on the insulating properties of the SiO_2_ film, we performed *I*–*V* measurements. The current density–electric field (*J*–*E*) curves of the SiC MOS capacitors were shown in Figure 5. Compared to the SiO_2_/SiC sample, the SiO_2_/1 nm-Al_2_O_3_/SiC and SiO_2_/2 nm-Al_2_O_3_/SiC exhibit lower leakage currents, with the *E*_bd_ increasing to 10.9 MV/cm, 9.94 MV/cm, respectively. All three samples show typical Fowler–Nordheim (FN) tunneling characteristics. The expression for FN tunneling is as follows [39]:

(1) $$J=\frac{q^{3}E^{2}}{16\pi^{2}\hbar \varphi_{ox}}\exp\left[-\frac{4\sqrt{2m_{T}^{*}\varphi_{B}^{3/2}}}{3\hbar qE}\right]$$

where *q* is the elementary charge, $m_{T}^{*}$ is the effective mass of the electrons in the SiO_2_ film, and *ℏ* is the reduced Planck constant. By calculating the *J*–*E* data for the three samples, the results are shown in Table 1. We calculated the effective electron barrier heights for SiO_2_/SiC, SiO_2_/1 nm-Al_2_O_3_/SiC, and SiO_2_/2 nm-Al_2_O_3_/SiC, which are 2.15 eV, 2.51 eV, and 2.37 eV, respectively. Therefore, it can be concluded that the passivation layer effectively prevents the diffusion of harmful chemical species during the oxidation process of SiC, as previously reported in previous studies [29]. This helps improve the insulating properties of the SiO_2_ film and contributes to a more stable and improved interface structure. This also explains why the conduction band offset of SiO_2_/1 nm-Al_2_O_3_/SiC is closer to the theoretical value.

### 3.2. Thermal Stability of SiC MOS Capacitors

**Figure 6:**
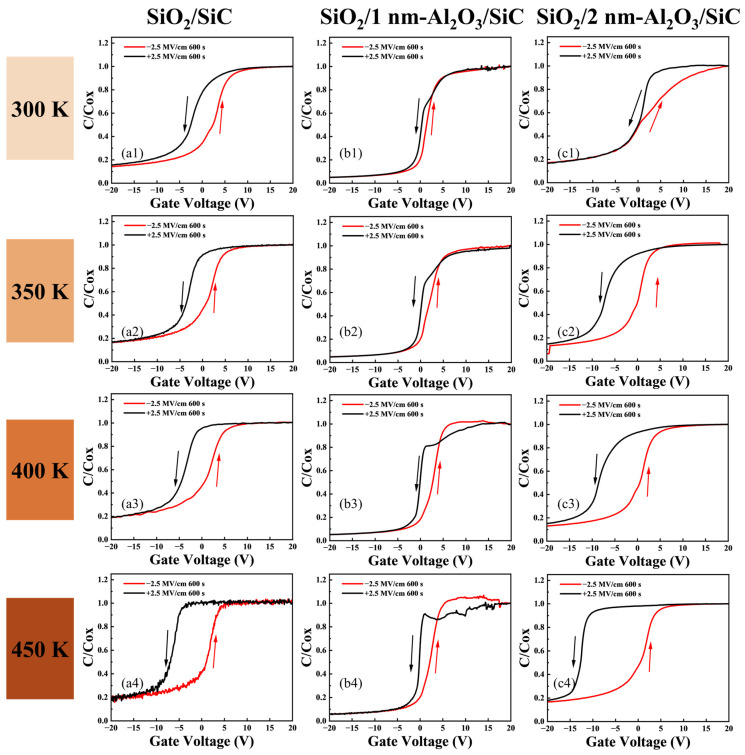
High-frequency *C*–*V* curves for SiC MOS capacitors after alternate PBS (2.5 MV cm^−1^, 600 s) and NBS (−2.5 MV cm^−1^, 600 s) for different samples at various temperatures, including SiO_2_/SiC at (**a1**) 300 K; (**a2**) 350 K; (**a3**) 400 K and (**a4**) 450 K; SiO_2_/1 nm-Al_2_O_3_/SiC at (**b1**) 300 K; (**b2**) 350 K; (**b3**) 400 K and (**b4**) 450 K and SiO_2_/2 nm-Al_2_O_3_/SiC at (**c1**) 300 K; (**c2**) 350 K; (**c3**) 400 K and (**c4**) 450 K.

In SiC MOS devices, the main sources of instability are typically considered to be the SiC/SiO_2_ interface and its near interface charge trapping, as well as the mobile ions in the gate oxide [40,41]. As shown in Figure 6, all the high-frequency (HF) *C*–*V* curves exhibit a counterclockwise hysteresis, indicating that the impact of trap charge trapping on the *C*–*V* drift in the samples is smaller than the effect of mobile ions [33].

**Figure 7:**
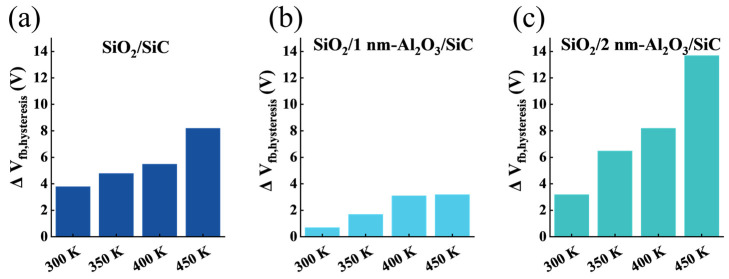
*V*_fb_ hysteresis in the high-frequency *C*–*V* curves for SiC MOS capacitors after alternate PBS (2.5 MV cm^−1^, 600 s) and NBS (−2.5 MV cm^−1^, 600 s) at temperatures ranging from 300 to 450 K for samples (**a**) SiO_2_/SiC; (**b**) SiO_2_/1 nm-Al_2_O_3_/SiC, and (**c**) SiO_2_/2 nm-Al_2_O_3_/SiC.

**Figure 8:**
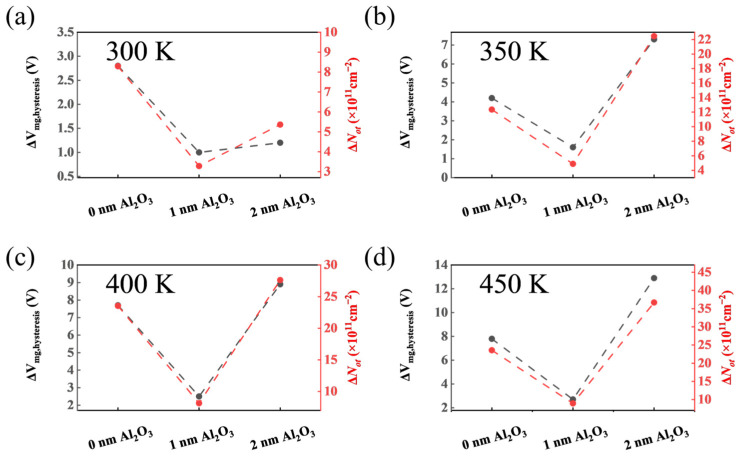
Δ*V*_mg_ hysteresis in the high-frequency *C*–*V* curves and areal density of Δ*N*_ot_ in the SiC MOS capacitors after alternate PBS (2.5 MV cm^−1^, 600 s) and NBS (−2.5 MV cm^−1^, 600 s) at (**a**) 300 K; (**b**) 350 K; (**c**) 400 K and (**d**) 450 K.

We also studied the changes in Δ*V*_fb_ and Δ*V*_mg_ hysteresis under alternating PBTS and NBTS at temperatures of 300 K, 350 K, 400 K, and 450 K for samples with passivation layer thicknesses. As shown in Figure 7 and Figure 8, Δ*V*_fb_ and Δ*V*_mg_ were extracted using midgap capacitance and flat-band capacitance as reference points. It was observed that in all three samples, the HF *C*–*V* curve hysteresis of SiO_2_/1 nm-Al_2_O_3_/SiC was relatively small at different temperatures, which demonstrates that the Al_2_O_3_ passivation layer with ten cycles reduces the traps near the interface, thereby improving the stability of BTS. Combined with the *D*_it_ results, it can be inferred that the 10c Al_2_O_3_ layer effectively prevents low-temperature oxidation of SiC and reduces interface traps, thereby improving the interface quality and the *V*_fb_ stability of the SiC MOS capacitors. As the passivation cycle reaches 20c, the Δ*V*_fb_ and Δ*V*_mg_ of SiO_2_/2 nm-Al_2_O_3_/SiC significantly increase. This increased hysteresis behavior indicates that the excessive Al_2_O_3_ passivation layer induces additional activated traps under high-temperature stress.

## 4. Conclusions

In this study, a SiO_2_/4H-SiC gate dielectric with low interface state density and high stability was successfully fabricated. A novel process was employed, in which an ultra-thin passivation layer of Al_2_O_3_ was deposited on the SiC surface using ALD. The Al_2_O_3_ layer protected the SiC surface by preventing the diffusion of harmful chemical species during the oxidation process. Si was then deposited atop the Al_2_O_3_ layer and re-oxidized to form a high-quality gate oxide layer. We systematically investigated the influence of the Al_2_O_3_ passivation layer on interface properties and optimized the fabrication method. Compared to samples without the Al_2_O_3_ passivation layer, the SiO_2_/1 nm-Al_2_O_3_/SiC MOS capacitor demonstrated a significant reduction in *D*_it_. Long-term high-temperature stability was assessed via flat-band voltage bias stress tests, revealing that the Al_2_O_3_ layer effectively suppressed the formation of near interface oxide traps and enhanced the stability of the oxide layer. This method offers a novel avenue for overcoming the performance bottlenecks of SiC MOSFETs.

## Figures and Tables

**Table 1:** Comparison of barrier height and breakdown field for the three 4H-SiC MOS structures.

	Theoretical [28]	SiO_2_/SiC	SiO_2_/1 nm-Al_2_O_3_/SiC	SiO_2_/2 nm-Al_2_O_3_/SiC
*φ*_B_ (eV) @25 °C	2.7	2.15	2.51	2.37
*E*_bd_ (MV cm^−1^)	10	9.64	10.9	9.94

## Data Availability

The data that support the findings of this study are available from the corresponding author, Hong-Ping Ma, upon reasonable request.
